# Lack of Plasma Protein Hemopexin Results in Increased Duodenal Iron Uptake

**DOI:** 10.1371/journal.pone.0068146

**Published:** 2013-06-27

**Authors:** Veronica Fiorito, Simonetta Geninatti Crich, Lorenzo Silengo, Silvio Aime, Fiorella Altruda, Emanuela Tolosano

**Affiliations:** 1 Molecular Biotechnology Center, University of Torino, Torino, Italy; 2 Department of Molecular Biotechnology and Health Sciences, University of Torino, Torino, Italy; Medical University Innsbruck, Austria

## Abstract

**Purpose:**

The body concentration of iron is regulated by a fine equilibrium between absorption and losses of iron. Iron can be absorbed from diet as inorganic iron or as heme. Hemopexin is an acute phase protein that limits iron access to microorganisms. Moreover, it is the plasma protein with the highest binding affinity for heme and thus it mediates heme-iron recycling. Considering its involvement in iron homeostasis, it was postulated that hemopexin may play a role in the physiological absorption of inorganic iron.

**Methods and Results:**

Hemopexin-null mice showed elevated iron deposits in enterocytes, associated with higher duodenal H-Ferritin levels and a significant increase in duodenal expression and activity of heme oxygenase. The expression of heme-iron and inorganic iron transporters was normal. The rate of iron absorption was assessed by measuring the amount of ^57^Fe retained in tissues from hemopexin-null and wild-type animals after administration of an oral dose of ^57^FeSO_4_ or of ^57^Fe-labelled heme. Higher iron retention in the duodenum of hemopexin-null mice was observed as compared with normal mice. Conversely, iron transfer from enterocytes to liver and bone marrow was unaffected in hemopexin-null mice.

**Conclusions:**

The increased iron level in hemopexin-null duodenum can be accounted for by an increased iron uptake by enterocytes and storage in ferritins. These data indicate that the lack of hemopexin under physiological conditions leads to an enhanced duodenal iron uptake thus providing new insights to our understanding of body iron homeostasis.

## Introduction

The strong interest on iron nutrition and metabolism in both developing and developed nations arises from the need to find a remedy to the widely diffused metabolic disorders of iron deficiency and overload. Several interdisciplinary studies of the various aspects of iron nutrition, physiology, and biochemistry have been carried out. Particular attention has been devoted to studies about dietary and physiologic factors that modulate the efficiency of iron absorption with the aim of elucidating molecular mechanisms of intestinal absorption of iron. The purpose is to formulate diets and dietary practices that enhance iron availability and to unravel the precise pathways and general features of intestinal iron absorption mechanism. Despite many years of intense studies, many of these aspects are still speculative and hypothetical.

Dietary iron absorption can be divided into intestinal uptake (i.e., transport across the apical membrane of enterocytes) and transfer (i.e., translocation through the cytoplasm and across the basolateral membrane into the portal circulation). Anyway, consensus has not yet been reached on the comprehensive molecular mechanisms involved in iron passage into, across, and out of the mucosal epithelial cells.

In mammals, the majority of iron is present as hemoglobin in erythrocytes. The phagocytosis of senescent erythrocytes mediated by macrophages ensures that a significant portion of the iron is recycled. Nevertheless, a certain amount of iron is daily lost through epithelial exfoliation, thus requiring compensation by dietary iron absorption through duodenal enterocytes. In the absence of important pathologies, the body needs approximately 1 mg of iron per day to maintain iron balance. Nonheme iron exists in two main forms, Fe(III) (the ferric form) and Fe(II) (the ferrous form). Most dietary iron is nonheme iron, generally found in foods of vegetal origin. Before absorption through the divalent metal transporter 1 (DMT1), Fe(III) in the diet must be reduced to Fe(II) at the apical surface of enterocytes by the ferrireductase duodenal cytochrome-b (Dcytb). Once in the cytosol, iron can be stored in ferritin (Ft) or exported. The protein, poly (rC)-binding protein 1 (PCBP1) is involved in the translocation pathway of iron to the iron storage Ft protein, while at the basolateral membrane iron is transported out of the enterocyte into the portal blood circulation by the iron-export protein ferroportin 1 (Fpn1) [1]. Iron exported by Fpn1 is then oxidised by hephaestin (Heph) and bound by transferrin (Tf) in the circulation. The transferrin receptor (TfR1), located at the basal membrane of enterocytes, could eventually take up the circulating Tf-bound iron, transporting it back in duodenal enterocytes.

Another source of dietary iron is heme. Heme results from the breakdown of hemoglobin and myoglobin found in meat products. Heme represents the most important source of dietary iron in meat-eating animals, accounting for by one-third of ingested iron in Western diets and up to two-thirds of absorbed body iron. Although the molecule(s) mediating duodenal brush–border enterocyte heme uptake has not yet been clearly identified, there is considerable evidence to suggest that uptake occurs via a receptor-mediated endocytotic pathway or by the proton-coupled folate transporter/heme carrier protein-1 (PCFT/HCP1), expressed at high levels in the duodenum [1]. Once in the cytosol, heme is catabolised by heme oxygenase (HO), giving rise to iron, carbon monoxide and biliverdin. Moreover, the enterocytes express some heme exporters, such as ATP-binding cassette sub-family G member 2 (ABCG2) [2] and feline leukaemia virus subgroup C receptor 1a (FLVCR1a) [3,4], although their function in duodenal heme metabolism has not been described so far. Inside the enterocytes heme controls several mechanisms, acting both as a prosthetic group in hemoproteins, such as cytochromes, and as a regulator of gene transcription.

The regulation of iron absorption in the intestine is finely tuned in response to iron need in the organism and to iron deposits in body tissues. Indeed, the process is adjusted according to both the global body iron status and the local iron status in the absorptive enterocytes. A complex network of proteins that responds to and integrates systemic and local signals is responsible for this homeostatic regulation of dietary iron assimilation, ensuring sufficient iron uptake and avoiding toxic iron accumulation.

The hepatic hormone hepcidin (Hepc), the hypoxia inducible transcription factors (HIFs), the iron regulatory proteins (IRPs) and the iron storage proteins ferritins are the main factors for the regulation of systemic iron homoeostasis and cellular local iron balance, respectively [5,6]. Hepc and HIF, in particular, have been demonstrated to play a crucial role in the modulation of iron uptake from dietary sources and transfer to the organism to meet body iron requirements.

Hepc is a negative regulator of the Fpn1 protein located on the basolateral membrane of enterocytes [7]. Hepc synthesis is positively regulated by iron stores and Tf saturation, the latter reflecting a steady state between iron influx into plasma and iron uptake and utilization by the bone marrow and other tissues. In iron deficiency or in case of high iron demand from the erythropoietic compartment, such as during hemolysis, hepatic Hepc production decreases, allowing enterocyte Fpn1 levels to increase, thus facilitating basolateral export of iron. Moreover, in the presence of iron deficiency, the liver produces and secretes into the circulation more apo-Tf and ceruloplasmin, both of which favour the movement of iron into the portal blood. Besides its regulatory role of body iron stores in response to erythropoietic iron demand, Hepc is also important in inflammatory events to decrease iron release in plasma, thus limiting iron availability to pathogens [5].

Regarding HIFs, evidence has been gained to prove that they act as central mediators of cellular adaptation to critically low oxygen levels in both normal and compromised tissues. Several of their target genes are involved in iron homeostasis, reflecting the molecular links between oxygen homeostasis and iron metabolism [8–10].

HIFs allow the cells of peripheral tissues to sense the oxygen delivered by erythrocytes in order to modulate iron supply to the bone marrow for erythropoiesis. Furthermore, iron is a necessary cofactor in the post-translational modification of HIFs, thus explaining how HIFs, other than oxygen, can also sense iron and consequently regulate the expression of target genes involved in iron handling [11].

Hemopexin (Hx) is a glycoprotein whose major function has been so far described during hemolysis or systemic massive heme overload [12,13]. In these conditions, Hx binds and transports heme to hepatocytes, where heme can be efficiently catabolised [14]. Hx or heme-Hx complexes regulate gene expression [15,16], act as signaling molecules and exert important functions in the nervous and immune system [12]. As Hepc, Hx is a plasma protein released by liver, its production is regulated during hemolysis and inflammatory events and it is considered an acute phase protein [13]. For this reason, additional functions for Hx in the control of iron trafficking can be postulated.

This work describes a role for Hx in modulating iron uptake by duodenum under physiologic conditions.

## Materials and Methods

### Animals

Wild-type and Hx-null mice in the 129Sv genetic background were fed on a standard diet (4RF25 GLP, Mucedola, Settimo Milanese, Milano, Italy) containing 8 mg/kg and 292 mg/kg heme-iron and inorganic iron respectively, and received water ad lib. All experiments were approved by the animal studies committee of the University of Torino, Italy.

### Tissue iron measurement

^57^Fe isotope and total Fe were determined using inductively coupled plasma mass spectrometry (ICP-MS) (Element-2; Thermo-Finnigan, Rodano (MI), Italy) at medium mass resolution (M/ΔM ~4000). To this purpose, sample lysis was performed with 2 ml of concentrated HNO_3_ (70%) under microwave heating at 160°C for 20 minutes (Milestone MicroSYNTH Microwave labstation equipped with an optical fiber temperature control and HPR-1000/6M six position high-pressure reactor, Bergamo, Italy). After lysis, the volume of each sample was brought to 3 ml with ultrapure water and the sample was analyzed by ICP-MS. A natural abundance iron standard solution was analyzed during sample runs in order to check changing in the systematic bias. The calibration curve was obtained using four iron absorption standard solutions (Sigma-Aldrich) in the range 0.2–0.05 µg/ml.

Tissue iron was also measured by the BPS-based colorimetric method and by DAB (methanol 3,3 diamino-benzidine) enhanced Perls’ stain, as previously reported [17].

### ^57^Fe and ^57^Fe-heme absorption

For ^57^Fe absorption analyses, the stable iron isotope ^57^Fe (^57^Fe at 94% enrichment; Frontier Scientific Inc., Logan, Utah USA) was used as tracer.

A 0.4 mol/L solution of ^57^FeSO_4_ has been prepared by overnight dissolution of 22.85 g ^57^Fe/L in 0.4 mol/L H_2_SO_4_ (Sigma Aldrich, Milano, Italy). The obtained ^57^FeSO_4_ solution was stored at 4°C. Before its use, 87.7 mg sucrose and 0.83 mg ascorbic acid per mg iron were added to the ^57^FeSO_4_ solution to yield to a final concentration of ^57^Fe of 20 mmol/L, ascorbic acid of 5.38 mmol/L and sucrose of 10%, respectively.

As negative control, an analogous solution without tracer was prepared.

Both the ^57^FeSO_4_-labelled and the control solution were adjusted to pH=7 by adding the required volume of 1 mol/L NaOH.

For ^57^Fe-heme absorption analyses, 10mg of ^57^Fe(III) Protoporphyrin IX chloride (Frontier Scientific Inc., Logan, Utah USA) were dissolved in the required volume of DMSO 100% to yield a final concentration of 20 mmol/L. The obtained solution was stored at 4°C.

To assess the in vivo absorption of ^57^FeSO_4_ or ^57^Fe-heme, 20 µl of ^57^FeSO_4_-labelled solution (correspondent to 22.8 µg ^57^Fe) or 20 µl of ^57^Fe-heme labelled solution (correspondent to 22.8 µg ^57^Fe contained in 260.8 µg ^57^Fe-heme) were orally administered to overnight fasted mice. Control mice received vehicle solution. During the experiment mice received water ad lib. Tissues were collected at different times after the administration. Control mice represented the “0” time point of the experiment.

The amount of ^57^Fe retained by the tissue upon the administration of ^57^FeSO_4_-labelled or ^57^Fe-heme labelled solutions was determined by inductively coupled plasma mass spectrometry (ICP-MS) and expressed as μg of ^57^Fe per g of wet tissue, taking into account the amount of naturally occurring ^57^Fe.

The percentage natural abundance of ^57^Fe in tissues of wild-type and Hx-null animals was checked before ^57^Fe and ^57^Fe-heme absorption analyses, resulting comparable in the two groups (Figure S1).

Further details on the experimental procedure are reported in [18].

### Heme oxygenase activity

HO activity was evaluated in tissue microsomal fractions by measuring bilirubin production. Briefly, frozen tissue samples were prepared by homogenization in hypotonic buffer (10 mmol/L Tris-HCl buffer pH 7.4, 2 mmol/L MgCl_2_) with protease inhibitors (aprotinin, leupeptin, pepstatin; Cocktail Tablets, Roche Diagnostics). After 15 minutes incubation on ice, samples were sonicated and the homogenates were then adjusted to 0.25 mol/L sucrose. After centrifugation for 10 minutes at 1000g, the supernatant was removed and centrifuged for an additional 15 minutes at 12000g before being ultracentrifuged at 33000 rpm for 1 hour. The supernatant was discarded and the microsomal pellet was used for HO activity measurement. The enzyme reaction method was used in a 200 µl mixture (prepared in potassium phosphate buffer 100 mmol/L, pH 7.4, 2mmol/L MgCl_2_) containing 150 µg microsomal proteins, 25 µmol/L hemin, 1 mmol/L NADPH, 2 mmol/L glucose-6-phosphate (G6P), 0.5 U G6P dehydrogenase, and 1 mg of rat liver cytosol proteins (33000 rpm supernatant) as a source of biliverdin reductase.

Rat liver supernatant was prepared fresh by homogenization in 0.1 mol/L sodium citrate buffer, pH 5, containing 10% glycerol, and centrifugation at 12000g for 15 minutes before ultracentrifugation at 33000 rpm for 1 hour.

Following 1 hour incubation in the dark at 37°C, the bilirubin generated was measured in a spectrophotometer at 464 nm and 530 nm. A control reaction containing all components except the protein extract was used for the spectrophotometric blank. The bilirubin was calculated by the difference in absorption between 464 and 530 nm. Results were expressed as pmol bilirubin/mg microsomal protein/h.

### Immunohistochemistry and Western blotting

Immunohistochemistry and Western blotting were performed according to standard procedures using antibodies against L- and H-ferritin [19], heme oxygenase (HO)-1 and -2 (Stressgen Bioreagents, Ann Arbor, MI, USA), ferroportin (Fpn) 1 [20], divalent metal transporter (DMT) 1 [21], transferrin receptor (TfR) 1 (Invitrogen, San Giuliano Milanese, Italy), proton-coupled folate transporter/heme carrier protein 1 (PCFT/HCP1) [22], and actin (Santa Cruz Biothecnology Inc., Santa Cruz, California, USA).

### Real Time PCR analysis

Total RNA was extracted using Purelink micro to midi RNA exctraction kit (Invitrogen, San Giuliano Milanese, Italy) and analyzed by quantitative real-time polymerase chain reaction (qRT-PCR). To this end, 1µg total RNA was transcribed into complementary DNA (cDNA) by M-MLV reverse transcriptase (Invitrogen, San Giuliano Milanese, Italy) and random primers (Invitrogen, San Giuliano Milanese, Italy). qRT-PCR was performed on a 7300 Real Time PCR System (Applied Biosystems, Monza, Italy) as previously reported [16]. qRT-PCR analysis was performed using the Universal Probe Library system (Roche). Primers and probes were designed using the ProbeFinder software (www.roche-applied-science.com). Transcript abundance, normalized to 18S mRNA expression, is expressed as a fold increase over a calibrator sample.

### Statistical Analysis

Results were expressed as mean ±SEM. Statistical analyses were performed using two-way analysis of variance followed by the Bonferroni correction for multiple group comparisons. An unpaired Student’s *t*-test was used when only two groups were compared. A *P* value of less than 0.05 was regarded as significant.

## Results

### Hx deficiency results in increased iron deposits in the duodenum

To assess the role of Hx in the iron absorption, iron content in duodenum and peripheral tissues of Hx-null and wild-type mice was evaluated by means of three different modalities.

**Figure 1:**
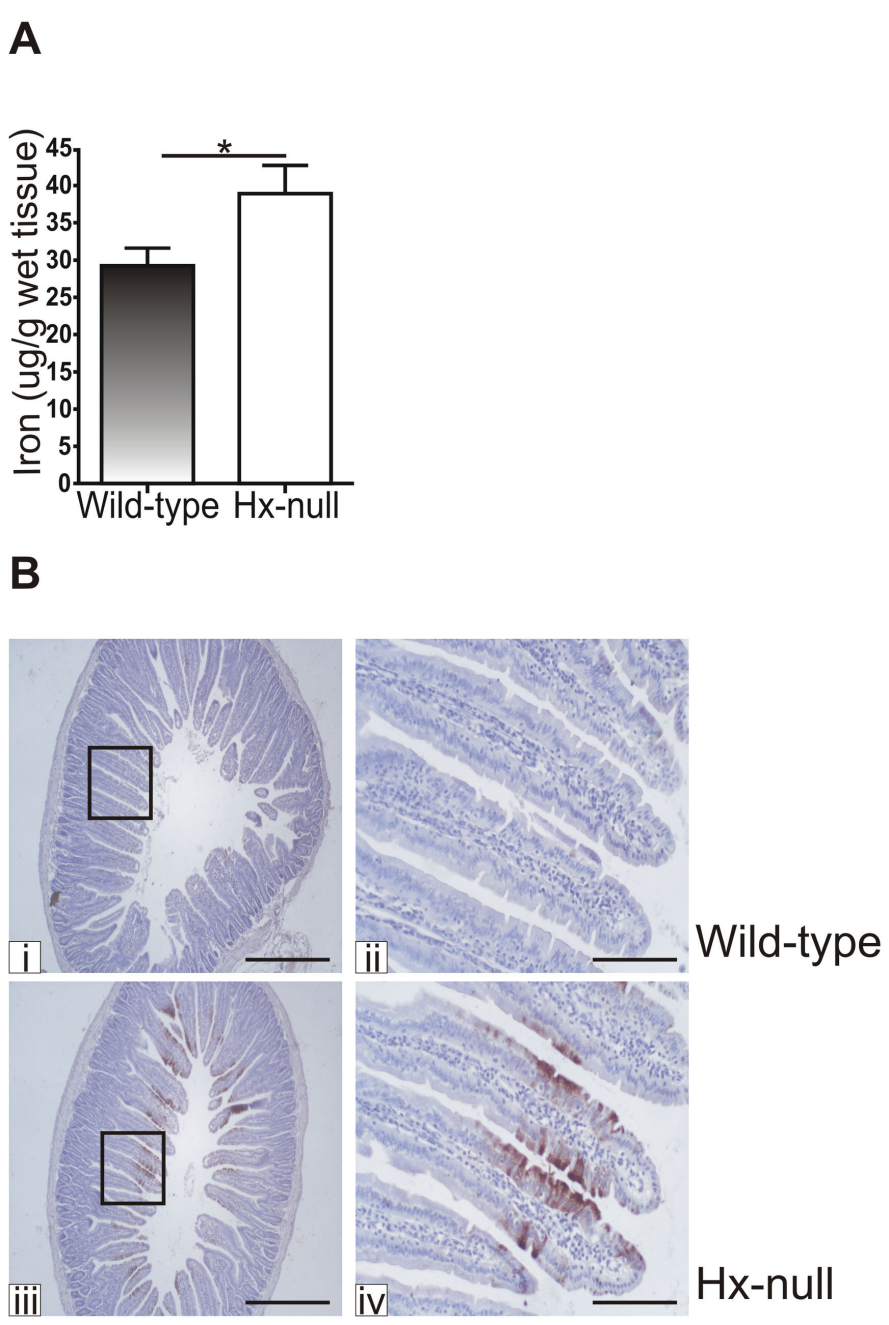
Hx deficiency results in increased iron deposits in the duodenum. (A) Total iron content in the duodenum of 2 month-old wild-type and Hx-null mice measured by ICP-MS. Values are expressed as μg iron/g wet tissue. Data represent mean ± SEM; n=10; * = P<0.05. (B) Duodenal sections of a wild-type mouse (i, ii) and an Hx-null mouse (iii, iv) at 2 months of age stained with DAB enhanced Perls’ reaction. Note the more intense staining in the duodenum of the Hx-null mouse. Bar i, iii = 500µm; bar ii, iv = 100µm.

First, duodenal iron content was determined by the BPS-based colorimetric method. A significant iron accumulation was detected in the duodenum of Hx-null mice compared to age-matched wild-type controls (Table 1), whereas liver, spleen, kidney and serum iron levels were comparable in the two groups of mice [17]. Duodenal iron content was similar in 2 and 6 month-old Hx-null mice. This observation supports the view that the detected amount of iron likely represents the maximal iron capacity of duodenal mucosa, a tissue with a rapid turnover. The increased iron content in the duodenum of Hx-null mice was confirmed by ICP-MS measurements (Figure 1A). Furthermore, iron staining by DAB enhanced Perls’ reaction on tissue sections clearly showed higher iron deposits in Hx-null mice duodenum than in wild-type controls (Figure 1B).

**Table 1:** Hx deficiency results in increased iron deposits in the duodenum.

	2 months old mice	6 months old mice
Wild-type	91.07 ± 12.06 (n=7)	129.0 ± 13.40 (n=4)
Hx-null	221.5 ± 26.08 (n=12)	252.4 ± 36.19 (n=4)
P value	0.0020	0.0187

Measurement of tissue iron in the duodenum of 2 and 6 months-old wild-type and Hx-null mice. Values are expressed as μg/g dry tissue. Note the significantly higher iron levels in the Hx-null mice duodenum compared to wild-type mice at both ages. Comparison between mice of the same genotype at 2 and 6 months of age did not reveal significant differences.

**Figure 2:**
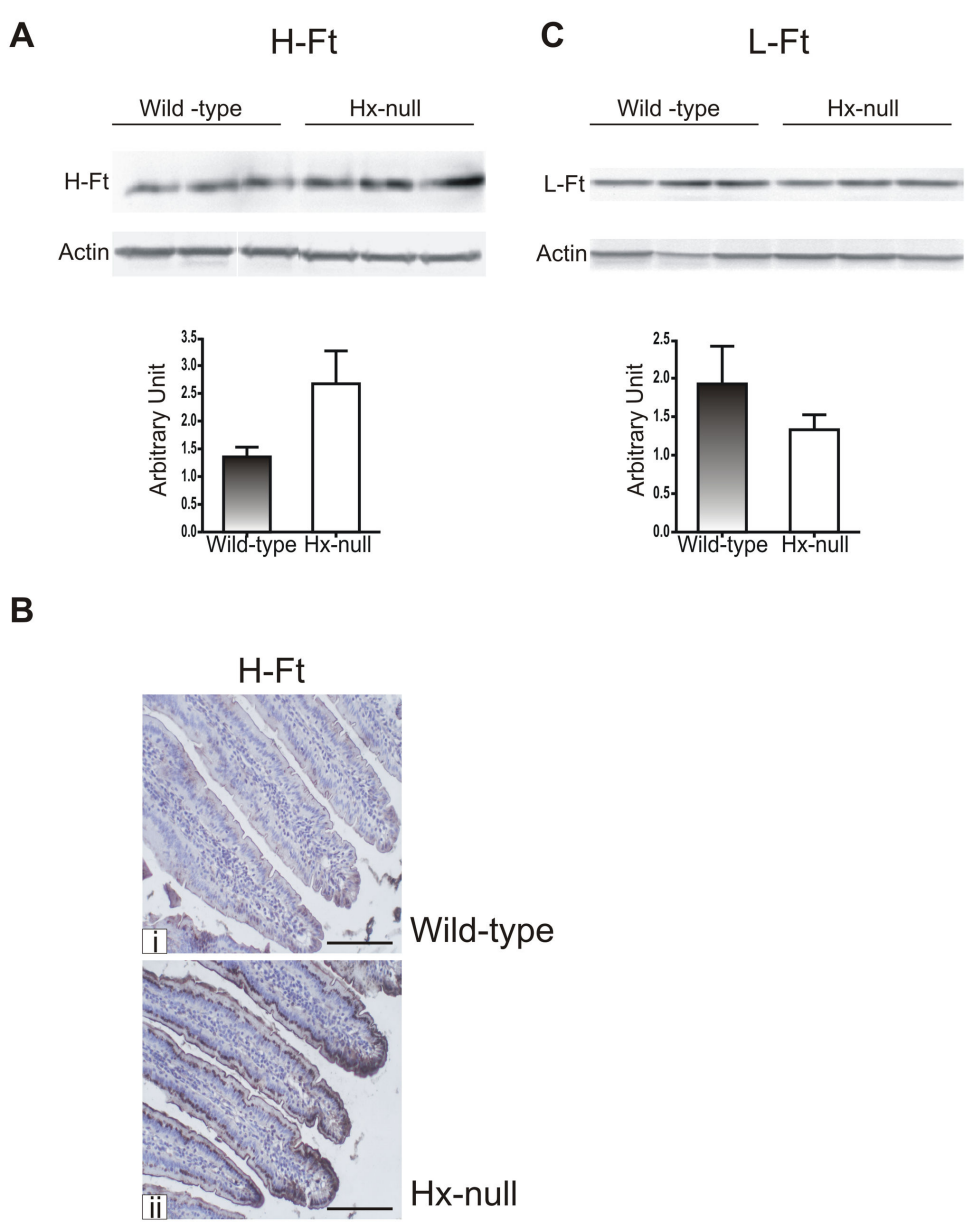
Increased H-Ft expression in Hx-null mice duodenum. Representative Western blots of H-Ft (A) and L-Ft (C) expression in the duodenum of wild-type and Hx-null mice. Band intensities were measured by densitometry and normalized to actin expression. Densitometry data represent mean ± SEM; n=3 for each genotype. (B) Duodenal sections of a wild-type mouse (i) and an Hx-null mouse (ii) processed by immunohistochemistry with an anti-H-Ft antibody. The H-Ft-positive signal is increased in the duodenum of the Hx-null animal. Bar= 100µm.

Iron loading in Hx-null animals was associated with an increase in H-ferritin (H-Ft) expression in duodenal enterocytes (Figure 2A, B), whereas L-Ft level did not show a significant change (Figure 2C).

### Hx deficiency does not affect the expression of duodenal iron transporters

**Figure 3:**
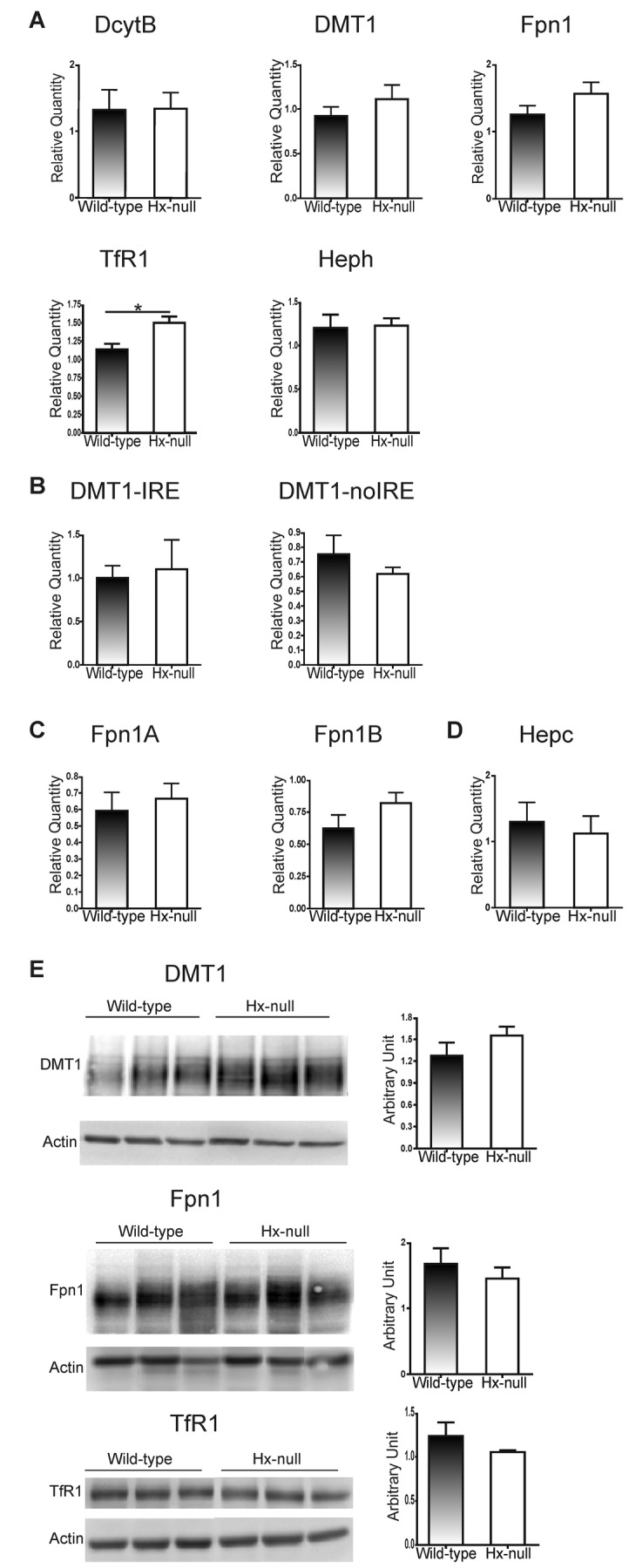
Hx deficiency does not affect the expression of duodenal iron transporters. (A) qRT-PCR analysis of DcytB, DMT1, Fpn1, TfR1 and Heph expression in the duodenum of wild-type and Hx-null mice. These assays do not discriminate between the different DMT1 and Fpn1 isoforms. The results of specific qRT-PCR assays for DMT1-IRE and DMT1-noIRE expression and for Fpn1A and Fpn1B expression are shown in (B) and (C), respectively. (D) qRT-PCR analysis of Hepc expression in the liver of wild-type and Hx-null mice. In A-D, transcript abundance, normalized to 18S RNA expression, is expressed as a fold increase over a calibrator sample. Data represent mean ± SEM, n= 6 for each genotype. (E) Representative Western blots of DMT1, Fpn1 and TfR1 expression in the duodenum of wild-type and Hx-null mice. Band intensities were measured by densitometry and normalized to actin expression. Densitometry data represent mean ± SEM; n=3 for each genotype. Results shown are representative of 3 independent experiments.

To get more insight into the molecular mechanisms underlying iron overload, the expression of DcytB, DMT1, Fpn1, TfR1 and Heph in the duodenum has been assessed. qRT-PCR analysis showed that DcytB, DMT1, Fpn1 and Heph mRNA levels were not altered in Hx-null mice compared to wild-type animals, while TfR1 mRNA level was higher in Hx-null mice than in controls (Figure 3A). Since duodenum cells express two types of DMT1 and Fpn1 transcripts, that differ for the presence or absence of an Iron Responsive Element (IRE) sequence in their C-terminus or N-terminus [23,24] respectively, specific qRT-PCR assays able to discriminate between the isoforms were set up. The qRT-PCR results showed that both DMT1-IRE and DMT1-noIRE were expressed at comparable levels in the duodenum of Hx-null and wild-type mice (Figure 3B). An analogous result was found for IRE containing and not-containing Fpn1 transcripts (Fpn1A and Fpn1B in Figure 3C). Moreover, as Fpn1 is mainly regulated at post-transcriptional level by the hepatic hormone Hepc, the level of Hepc expression in the liver has been assessed. As shown in Figure 3D, Hepc expression did not result altered in Hx-null mice when compared to controls. Consistently with mRNA data, DMT1 and Fpn1 protein expression was similar in Hx-null and wild-type duodenum (Figure 3E). Interestingly, TfR1 protein showed a similar abundance in Hx-null and wild-type mice (Figure 3E), in contrast with the results obtained by measuring TfR1 transcript level.

### Hx deficiency results in enhanced heme catabolism in duodenum without an increased expression of duodenal heme transporters

As duodenal iron deposits in Hx-null mice could derive from altered heme catabolism and/or heme trafficking in enterocytes, the activity and expression of HO as well as the expression of heme transportes were evaluated.

**Figure 4:**
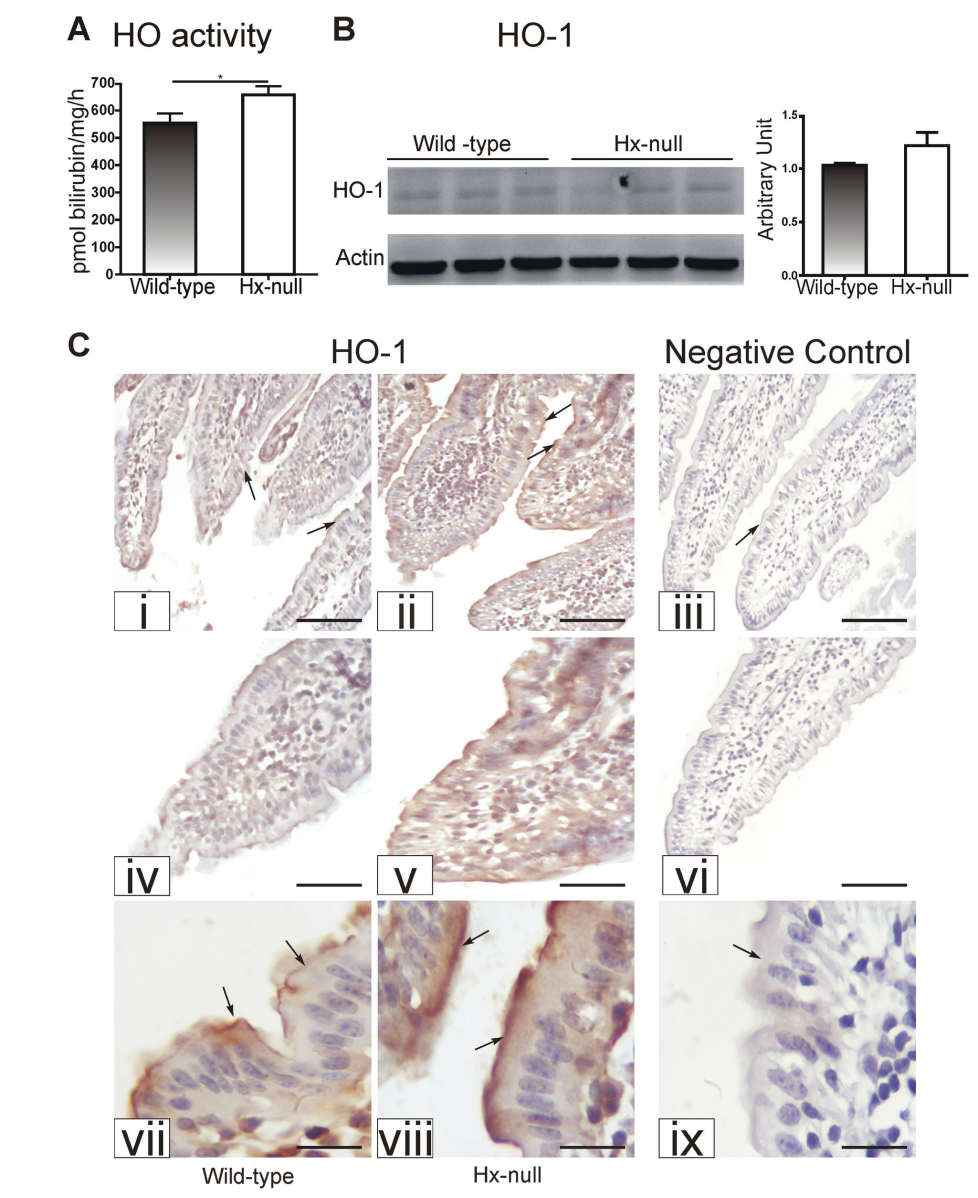
Hx deficiency results in enhanced heme catabolism in the duodenum. (A) HO activity in the duodenum of wild-type and Hx-null mice. Data represent mean ± SEM; n= 8 for each genotype. * = P<0.05. (B) Representative Western blot of HO-1 expression in the duodenum of wild-type and Hx-null mice. Band intensities were measured by densitometry and normalized to actin expression. Densitometry data represent mean ± SEM; n=3 for each genotype. (C) Sections of the duodenum of a wild-type mouse (i, iv, vii) and an Hx-null mouse (ii, v, viii) stained with an antibody to HO-1. Enlarged details of sections i, ii, iii are shown in iv, v, vi respectively The HO-1-positive signal was more intense in the Hx-null mouse than in the wild-type control (arrows) Sections on the right (iii, vi, ix) represent negative controls in which the primary antibody was omitted. Bar i, ii, iii = 100µm; bar iv, v, vi = 57 µm; bar vii, viii, ix = 20 µm.

HO activity was higher in the duodenum of Hx-null mice than in that of wild-type animals (Figure 4A). Western blot analysis on total duodenal lysates did not show differences in the expression of both HO-1 and HO-2 between Hx-null and wild-type mice (Figures 4B and S2A). Nevertheless, immunohistochemistry on duodenal sections showed an increased expression of HO-1 in duodenal villi of Hx-null mice (Figure 4B). In these animals, HO-1 immunoreactivity yielded a strong, continuous signal along the villi. At cellular level, HO-1 expression appeared higher in the apical cytoplasm of enterocytes, and decreased along their apical-basal axis (shown at high magnification in Figure 4B). On the contrary, in wild-type animals, the HO-1-positive signal along the villi appeared weaker and discontinuous whereas the subcellular pattern of expression was similar to that observed for Hx-null mice. As expected for a constitutive isoform, the HO-2 signal was similar in Hx-null and wild-type mice (Figure S2B).

**Figure 5:**
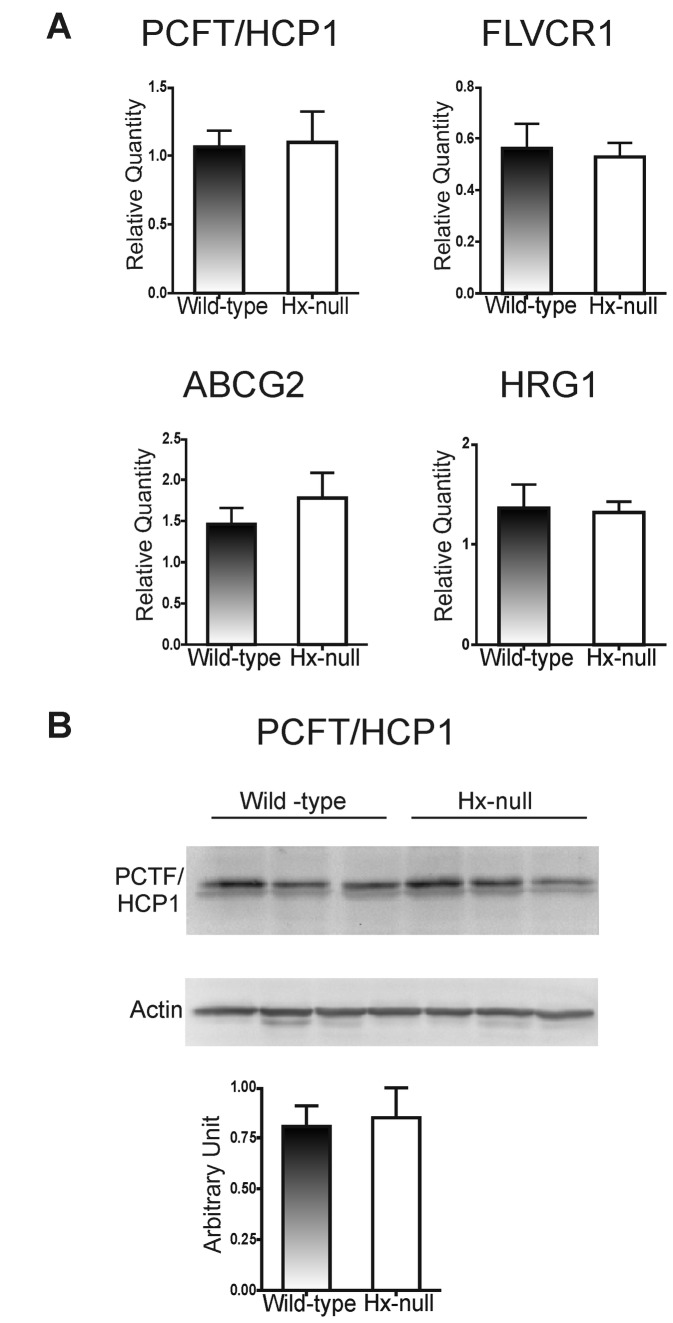
Hx deficiency does not affect the expression of duodenal heme transporters. (A) qRT-PCR analysis of PCFT/HCP1, FLVCR1, ABCG2 and HRG-1 expression in the duodenum of wild-type and Hx-null mice. Transcript abundance, normalized to 18S RNA expression, is expressed as a fold increase over a calibrator sample. Data represent mean ± SEM, n= 6 for each genotype. (B) Representative Western blot of PCFT/HCP1 expression in the duodenum of wild-type and Hx-null mice. Band intensities were measured by densitometry and normalized to actin expression. Densitometry data represent mean ± SEM; n=3 for each genotype. Results shown are representative of 3 independent experiments.

To assess whether the increased heme catabolism in the duodenum of Hx-null mice could be associated to an altered heme import from the intestinal lumen or to an altered export to plasma, it was decided to analyze the expression of PCFT/HCP1 and FLVCR1, the most well-characterized heme importer and exporter respectively. As shown in Figure 5A and 5B, PCFT/HCP1 and FLVCR1 mRNA and PCFT/HCP1 protein resulted expressed at similar levels in the duodenum of Hx-null and wild-type mice. In addition, when mRNA levels of other heme exporters were evaluated, it was found that the ATP-binding cassette, subfamily G, member 2 (ABCG2), as well as the intracellular heme transporter Heme Regulated Gene (HRG)-1 were unchanged in Hx-null duodenum (Figure 5A).

Thus, the increased heme catabolism in duodenum cells of Hx-null mice cannot be accounted for by an impaired expression of heme transporters.

### Hx deficiency results in an enhanced iron uptake in the duodenum

To assess the rate of iron absorption, an oral dose of ^57^FeSO_4_ or of ^57^Fe-labelled heme was administered to Hx-null and wild-type mice. ^57^Fe content in duodenum, liver, bone marrow and kidney was determined by inductively coupled plasma mass spectrometry (ICP-MS) analysis [18] at different times after the administration.

**Figure 6:**
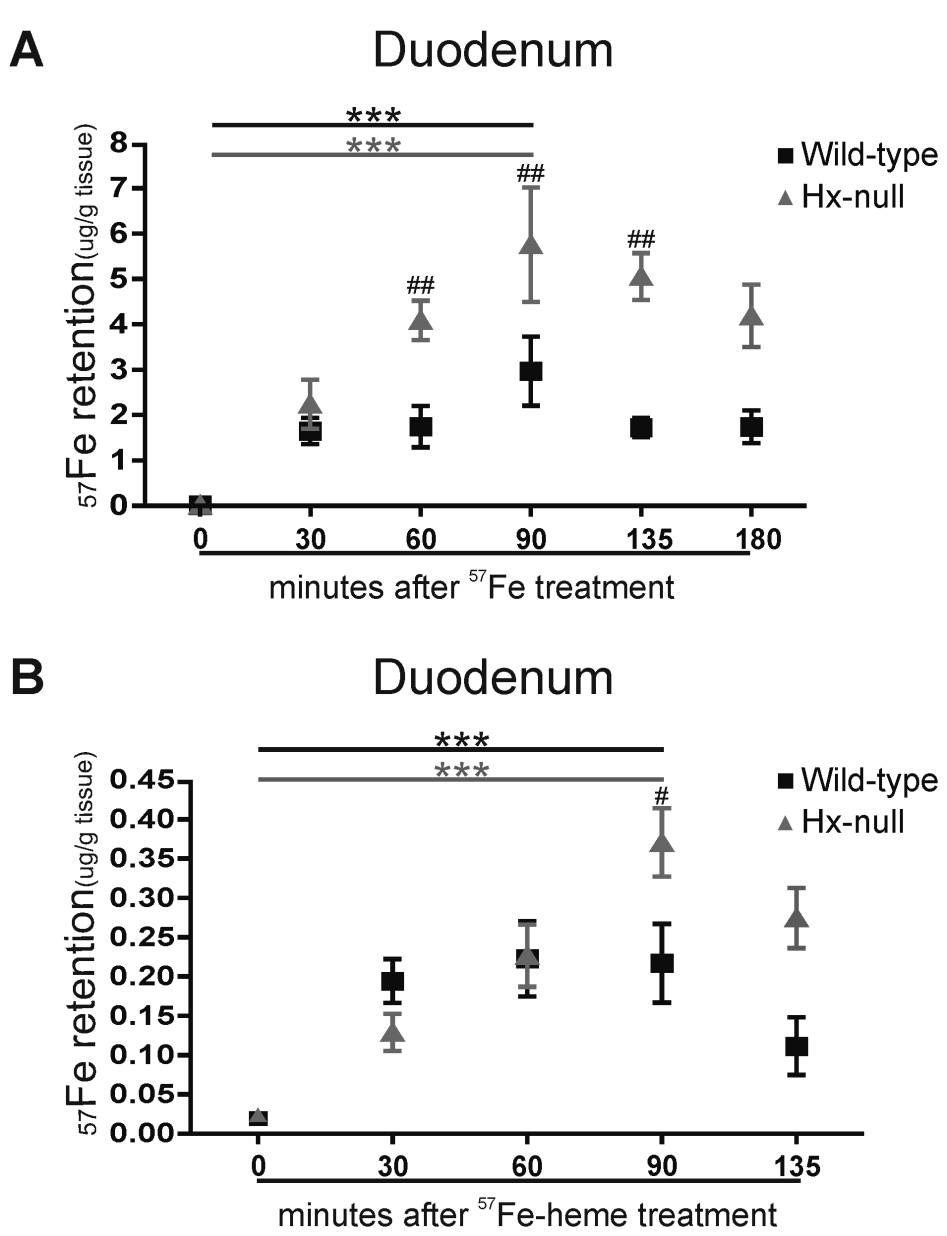
Hx deficiency results in enhanced iron uptake in the duodenum cells. (A) ^57^Fe retention in the duodenal mucosa of wild-type and Hx-null mice measured by ICP-MS 30, 60, 90, 135 and 180 minutes after oral administration of a solution containing 20 mmol/L ^57^FeSO_4_. Control mice were administered vehicle solution and represented the “0” time point of the experiment. Values are expressed as μg ^57^Fe/ g tissue. Data represent mean ± SEM; n=6 for each experimental point; *** = P<0.001 (comparing control mice with the corresponding group of ^57^FeSO_4_-administered mice), # # = P<0.01 (comparing the two genotypes). (B) ^57^Fe retention in the duodenal mucosa of wild-type and Hx-null mice measured by ICP-MS 30, 60, 90 and 135 minutes after oral administration of a solution containing 20 mmol/L ^57^Fe labelled heme (^57^Fe-heme). Control mice were administered vehicle solution and represented the “0” time point of the experiment. Values are expressed as μg ^57^Fe/ g tissue. Data represent mean ± SEM; n=6 for each experimental point; *** = P<0.001 (comparing control mice with the corresponding group of ^57^Fe-heme-administered mice), # = P<0.05 (comparing the two genotypes).

Thirty minutes after ^57^FeSO_4_ administration, a higher amount of ^57^Fe was detected in the duodenal mucosa of treated mice as compared with controls, and the quantity of ^57^Fe retained by duodenum further increased at ninety minutes after the oral administration. The amount of ^57^Fe retained in the mucosa was significantly higher in the duodenum of Hx-null mice than in that of wild-type animals (Figure 6A).

Similar results were obtained when ^57^Fe labelled heme (^57^Fe-heme) was orally administered (Figure 6B).

**Figure 7:**
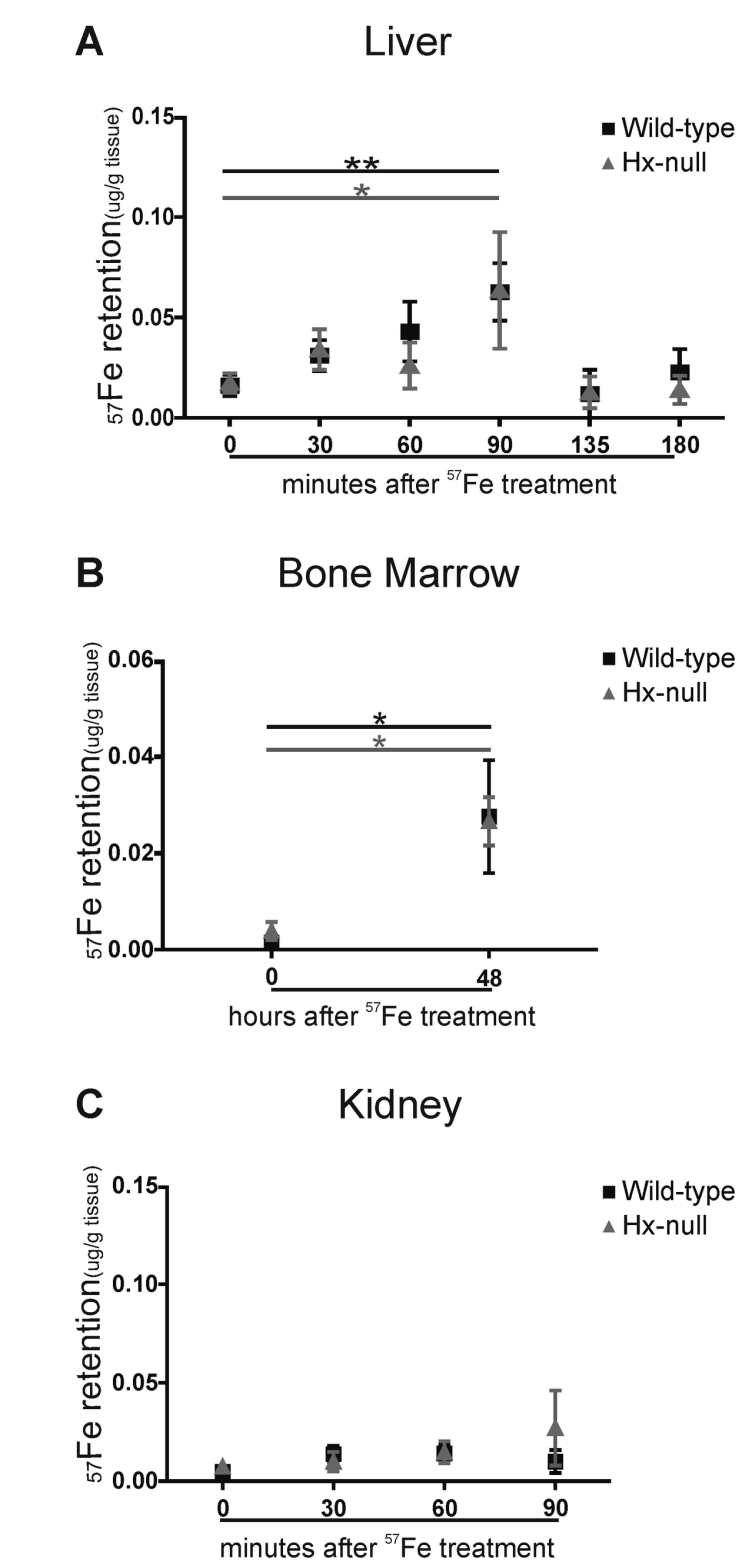
Hx deficiency does not affect iron transfer from the duodenum to other tissues. (A) ^57^Fe retention in the liver of wild-type and Hx-null mice measured by ICP-MS 30, 60, 90, 135 and 180 minutes after oral administration of a solution containing 20 mmol/L ^57^FeSO_4_. Control mice were administered vehicle solution and represented the “0” time point of the experiment. Values are expressed as μg ^57^Fe/ g tissue. Data represent mean ± SEM; n=6 for each experimental point; * = P<0.05, ** = P<0.01 (comparing control mice with the corresponding group of ^57^FeSO_4_-administered mice). (B) ^57^Fe retention in the bone marrow of wild-type and Hx-null mice measured by ICP-MS 48 hours after oral administration of a solution containing 20 mmol/L ^57^FeSO_4_. Control mice were administered vehicle solution and represented the “0” time point of the experiment. Values are expressed as μg ^57^Fe/ g tissue. Data represent mean ± SEM; n=10 for each experimental point; * = P<0.05. (C) ^57^Fe retention in the kidney of wild-type and Hx-null mice measured by ICP-MS 30, 60, and 90 minutes after oral administration of a solution containing 20 mmol/L ^57^FeSO_4_. Control mice were administered vehicle solution and represented the “0” time point of the experiment. Values are expressed as μg ^57^Fe/ g tissue. Data represent mean ± SEM; n=6 for each experimental point.

Analyses of liver and bone marrow showed comparable quantities of ^57^Fe in both Hx-null and wild-type mice after ^57^FeSO_4_ administration (Figure 7A, B). The transfer of ^57^Fe to the kidney, an organ only marginally involved in iron handling, was negligible in both genotypes (Figure 7C).

Collectively, these data showed that, upon an oral administration of ^57^FeSO_4_ or of ^57^Fe-labelled heme, iron accumulation in the duodenal mucosa of Hx-null mice was higher than in wild-type animals, whereas the ^57^Fe transport from the duodenal mucosa to peripheral tissues appeared unaffected. This demonstrates that the lack of Hx leads to an enhanced duodenal iron uptake.

## Discussion

The herein reported results demonstrate that the lack of Hx in plasma leads to an enhanced iron uptake in the duodenum, whereas iron transfer from duodenal mucosa to the body appears unaffected. The net result is an abnormal iron accumulation in enterocytes. Systemic iron balance is not affected by the lack of Hx as demonstrated by the normal Hepc expression, normal iron deposits in other tissues and normal hematological parameters in Hx-null mice [25].

The expression of iron transporters is not affected in duodenum cells of Hx-null mice despite the occurrence of increased iron deposits. Both DMT1-IRE and DMT1-noIRE as well as Fpn1A and Fpn1B are expressed at similar levels in Hx-null and wild-type mice. Moreover, TfR1 mRNA level is higher in Hx-null mice duodenum as compared with controls, but the amount of TfR1 protein is comparable in the two genotypes. Overall, these findings indicate that iron loading in the duodenum of Hx-null mice does not lead to significant changes in the activity of Iron Responsive Proteins (IRPs) [6]. This conclusion is further supported by the lack of induction of the expression of L-Ft in Hx-null duodenum, whereas the up-regulation of H-Ft appears to be controlled at a transcriptional level, likely by the increased amounts of dietary heme taken up by the Hx-null mice duodenum cells [26]. These results are quite surprising at the light of previous studies reporting a strong down-regulation of DMT1-IRE, together with an upregulation of L- and H-Ft, upon iron overload [27]. Nevertheless, in those experiments animals experienced an acute iron overload as they were challenged with a high iron dose (10 mg oral iron/rat). Moreover, DMT1 and Ft modulation after the iron dose was transient as the level of both proteins returned to basal values by 72 hours after iron administration. The situation encountered in the case of Hx-null mice appears different as they only ingested a limited quantity of iron with their daily diet. Our results indicate that a continuous and sustained iron uptake in duodenum is not sufficient to alter the activity of IRPs.

As far as TfR1 is concerned, it is interesting to note that the mRNA level of this receptor is up-regulated in Hx-null mice duodenum. This finding may indicate that, besides the IRE-IRPs system, different mechanisms could be involved in the regulation of TfR1 mRNA level, such as the modulation of transcription and/or mRNA stability. Nevertheless, the resulting protein level is comparable in Hx-null mice and wild-type controls, thus indicating that iron overload in Hx-null duodenum could not be ascribed to an altered expression of TfR1.

Another interesting feature deals with the increased HO-1 activity and expression observed in Hx-null mice as compared with wild-type animals.

Hx-null mice show enhanced ^57^Fe retention following ^57^Fe-heme administration, indicating increased duodenal heme uptake. The enhanced chronic dietary heme absorption in Hx-null mice could be responsible for the increased HO-1 activity and expression. Alternatively, the enhanced HO activity might derive from an impaired heme export through FLVCR1a or ABCG2 in the enterocytes of Hx-null mice. Although FLVCR1a and ABCG2 mRNA levels in Hx-null mice are comparable to those ones of wild-type mice, it could be hypothesized that the activity of these transporters is compromised in Hx-null mice. Notably, in vitro experiments have demonstrated that Hx interacts with FLVCR1a favouring cellular heme export [28].

Hx plasma levels are finely regulated during several pathological conditions. Hx is an acute phase protein and its plasma level raises during inflammatory events thanks to a transcriptional induction mediated by the cytokine interleukin 6 [13]. In these conditions Hx, by binding heme derived from dietary sources or hemolytic events, limits iron availability to pathogens thus inhibiting their growth. From the results obtained in this work it could be surmised that, in the presence of inflammation, Hx increase in plasma may have a role in inhibiting duodenal iron uptake, thus further contributing to limit bacterial expansion. On the other hand, Hx has been demonstrated to disappear from plasma in the presence of strong hemolytic processes, as the rate of its synthesis in the liver is not sufficient to replace its consumption [29]. Therefore, in the latter case, the depletion of Hx in the bloodstream might favour iron uptake in the duodenum, thus enhancing iron supply to erythropoietic compartments in a situation of high iron demand for red blood cells production. Moreover, the enhanced duodenal HO activity associated to Hx deficiency can further contribute to increase the amount of iron available to meet body iron requirement.

Interestingly, it has been reported that Hepc is upregulated by inflammation and strongly down-regulated during hemolysis [30] with the result of causing the blockage or the enhancement of iron export from duodenum cells, respectively. Thus, one may speculate that Hx and Hepc could cooperate to reduce iron absorption in case of inflammation and to enhance it during hemolysis. From this point of view the Hx-null mouse could be considered a model in which the axis Hx-Hepc is uncoupled (as Hx is absent while Hepc level is normal), thus justifying the presence of duodenal iron deposits in Hx-null mice.

The mechanism underlying the increase of duodenal iron uptake in the absence of Hx remains to be elucidated. As stated above, the expression of the most important duodenal inorganic iron and heme transporters is unaffected in Hx-null mice, thus suggesting the occurrence of alternative mechanisms other than transcriptional or translational regulation of these proteins.

An intriguing hypothesis is that Hx may modulate the activity of a specific transporter in duodenal cells. Of course, the fact that the regulation of iron uptake by Hx involves iron transporters exposed on the apical membrane of the enterocytes suggests that Hx can interact with a receptor activating a signalling pathway inside the absorptive cell. Consistently, the only known Hx receptor is LRP1/CD91 [31] which is ubiquitously expressed.

In a paper by Rish et al. [32] it was demonstrated that highly proliferative cells are characterized by a plasma membrane electron transport (PMET) that enables cells to transfer electrons from intracellular reductants, like NADH, to extracellular electron acceptors. Among electron acceptors, Rish et al. indicated the heme-Hx complex as one of the best physiological candidates for this function. A challenging idea is that plasma Hx, by generating heme-Hx complexes, might favour PMET contributing to maintain the typical steady state membrane potential of enterocytes. It might be possible that when Hx plasma levels are modified, as under pathological conditions, the general membrane potential of enterocytes may be altered. As the membrane potential is a pivotal regulator of iron and heme transporters activity, its modification can be associated to an enhanced or reduced heme and iron uptake/release. Further investigations are required to test these hypotheses.

In conclusion, the herein reported results show that the lack of Hx yields an increased duodenal iron uptake. This finding offers new perspectives for future studies aimed at investigating the reciprocal relationship between Hx and other hormones in the regulation of body iron homeostasis and possibly at identifying strategies to increase/reduce iron absorption in the therapy of metabolic disorders of iron deficiency and overload.

## Supporting Information

Figure S1 ^57^Fe natural abundance in tissues. Percentage of naturally occurring ^57^Fe in serum and tissues from wild-type and Hx-null animals determined by ICP-MS. Values are expressed as percentage of ^57^Fe respect to total iron. Data represent mean ± SEM, n= 10 for each genotype.
(TIF)

Figure S2 Hx deficiency does not affect duodenal HO-2 expression. (A) Representative Western blot of HO-2 expression in the duodenum of wild-type and Hx-null mice. Band intensities were measured by densitometry and normalized to actin expression. Densitometry data represent mean ± SEM; n=3 for each genotype. (B) Sections of the duodenum of a wild-type mouse (i, iv) and an Hx-null mouse (ii, v) stained with an antibody to HO-2. The HO-2-positive signal was comparable in the Hx-null mouse and in the wild-type control (arrows). Sections on the right (iii, vi) represent negative controls in which the primary antibody was omitted. Bar i, ii, iii = 100µm; bar iv, v, vi = 20 µm.
(TIF)
