# Supplementary figures and images for: Lack of Plasma Protein Hemopexin Results in Increased Duodenal Iron Uptake
Source: PLoS One. 2013 Jun 27;8(6):e68146. doi: 10.1371/journal.pone.0068146 (PMC3694894; doi:10.1371/journal.pone.0068146)

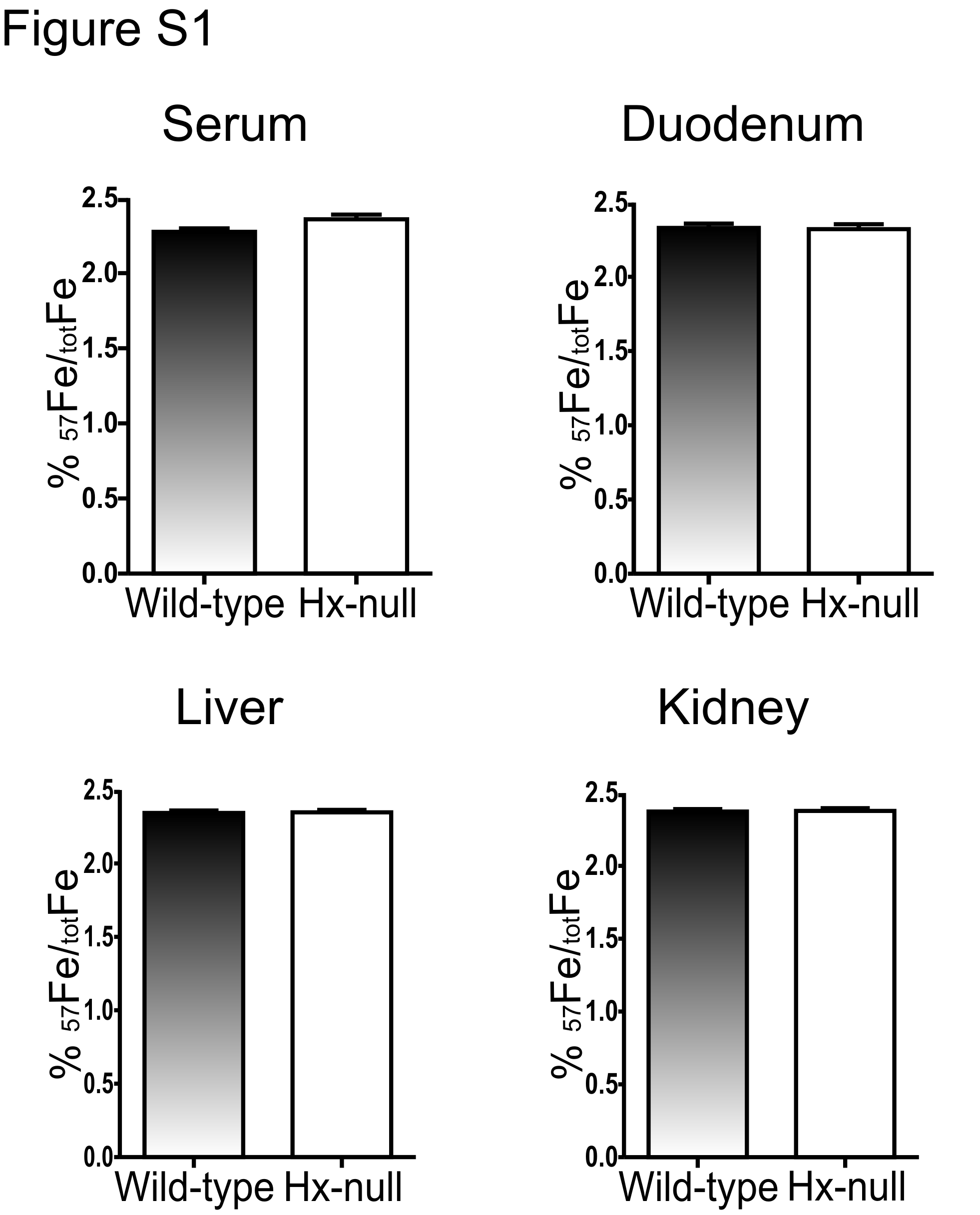

Supplement: Figure S1 — Percentage of naturally occurring 57Fe in serum and tissues from wild-type and Hx-null animals determined by ICP-MS. Values are expressed as percentage of 57Fe respect to total iron. Data represent mean ± SEM, n= 10 for each genotype. (TIF) [file pone.0068146.s001.tif]

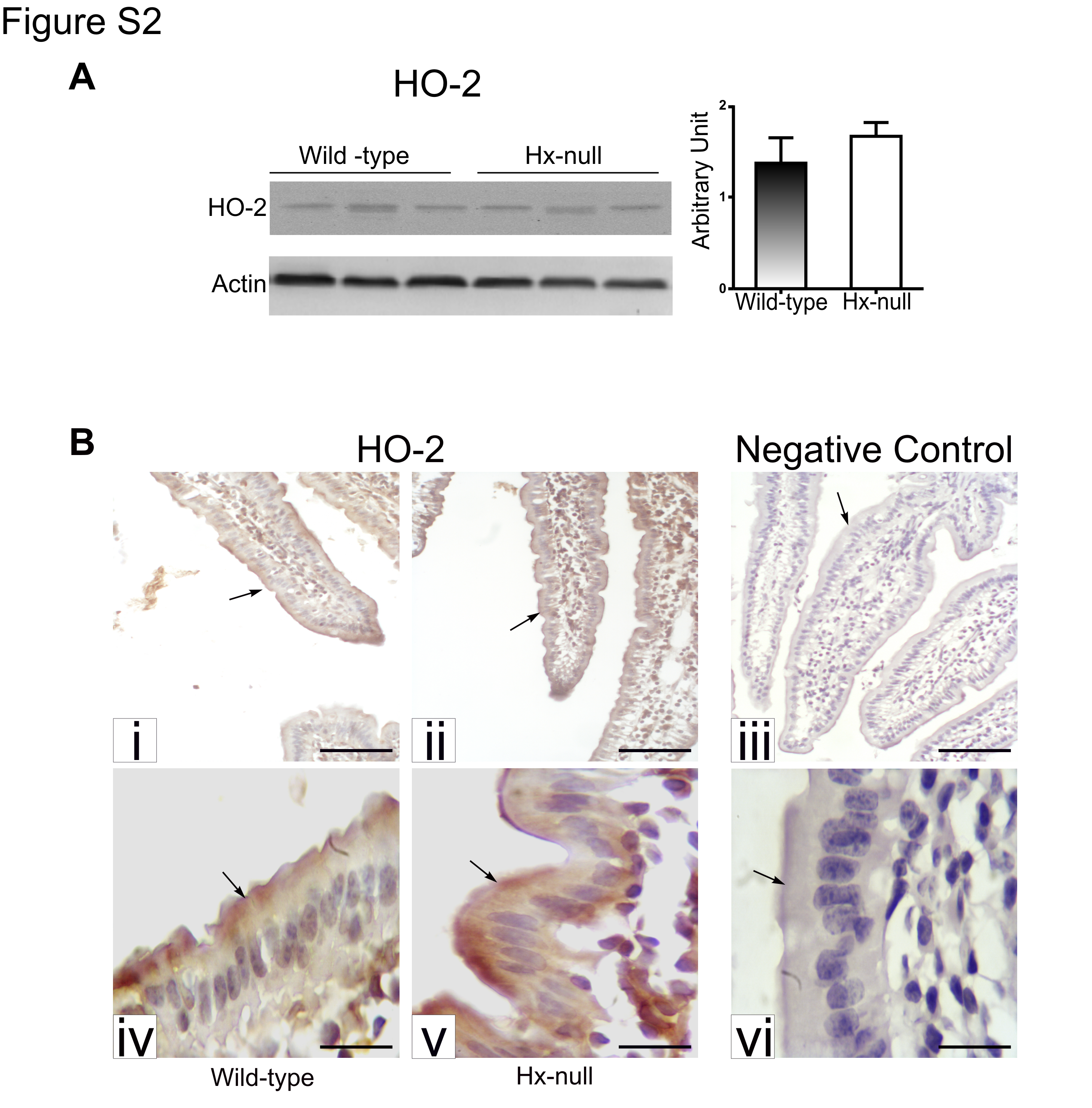

Supplement: Figure S2 — (A) Representative Western blot of HO-2 expression in the duodenum of wild-type and Hx-null mice. Band intensities were measured by densitometry and normalized to actin expression. Densitometry data represent mean ± SEM; n=3 for each genotype. (B) Sections of the duodenum of a wild-type mouse (i, iv) and an Hx-null mouse (ii, v) stained with an antibody to HO-2. The HO-2-positive signal was comparable in the Hx-null mouse and in the wild-type control (arrows). Sections on the right (iii, vi) represent negative controls in which the primary antibody was omitted. Bar i, ii, iii = 100µm; bar iv, v, vi = 20 µm. (TIF) [file pone.0068146.s002.tif]
